# Mitochondrial Morphology, Function and Homeostasis Are Impaired by Expression of an N-terminal Calpain Cleavage Fragment of Ataxin-3

**DOI:** 10.3389/fnmol.2018.00368

**Published:** 2018-10-10

**Authors:** Tina Harmuth, Caroline Prell-Schicker, Jonasz J. Weber, Frank Gellerich, Claudia Funke, Stefan Drießen, Janine C. D. Magg, Guido Krebiehl, Hartwig Wolburg, Stefanie N. Hayer, Stefan Hauser, Rejko Krüger, Ludger Schöls, Olaf Riess, Jeannette Hübener-Schmid

**Affiliations:** ^1^Institute of Medical Genetics and Applied Genomics, University of Tübingen, Tübingen, Germany; ^2^Centre for Rare Diseases, Tübingen, Germany; ^3^Graduate School of Cellular Neuroscience, University of Tübingen, Tübingen, Germany; ^4^Department of Neurology, University Hospital Magdeburg, Magdeburg, Germany; ^5^Center of Neurology and Hertie-Institute for Clinical Brain Research, University of Tübingen, Tübingen, Germany; ^6^Institute of Pathology and Neuropathology, University of Tübingen, Tübingen, Germany; ^7^German Center for Neurodegenerative Diseases, Tübingen, Germany; ^8^Department of Neurodegenerative Diseases, Hertie-Institute for Clinical Brain Research and Center of Neurology, University of Tübingen, Tübingen, Germany; ^9^Luxembourg Centre for Systems Biomedicine, University of Luxembourg, Centre Hospitalier de Luxembourg, Luxembourg, Luxembourg

**Keywords:** ataxin-3, lysosomal dysfunction, Machado-Joseph disease, mitochondrial dysfunction, S256 calpain cleavage fragment, Spinocerebellar Ataxia Type 3

## Abstract

Alterations in mitochondrial morphology and function have been linked to neurodegenerative diseases, including Parkinson disease, Alzheimer disease and Huntington disease. Metabolic defects, resulting from dysfunctional mitochondria, have been reported in patients and respective animal models of all those diseases. Spinocerebellar Ataxia Type 3 (SCA3), another neurodegenerative disorder, also presents with metabolic defects and loss of body weight in early disease stages although the possible role of mitochondrial dysfunction in SCA3 pathology is still to be determined. Interestingly, the SCA3 disease protein ataxin-3, which is predominantly localized in cytoplasm and nucleus, has also been associated with mitochondria in both its mutant and wildtype form. This observation provides an interesting link to a potential mitochondrial involvement of mutant ataxin-3 in SCA3 pathogenesis. Furthermore, proteolytic cleavage of ataxin-3 has been shown to produce toxic fragments and even overexpression of artificially truncated forms of ataxin-3 resulted in mitochondria deficits. Therefore, we analyzed the repercussions of expressing a naturally occurring N-terminal cleavage fragment of ataxin-3 and the influence of an endogenous expression of the S256 cleavage fragment *in vitro* and *in vivo*. In our study, expression of a fragment derived from calpain cleavage induced mitochondrial fragmentation and cristae alterations leading to a significantly decreased capacity of mitochondrial respiration and contributing to an increased susceptibility to apoptosis. Furthermore, analyzing mitophagy revealed activation of autophagy in the early pathogenesis with reduced lysosomal activity. In conclusion, our findings indicate that cleavage of ataxin-3 by calpains results in fragments which interfere with mitochondrial function and mitochondrial degradation processes.

## Introduction

Generation of free radicals and deficiencies in energy supply, calcium buffering, or regulation of apoptosis contribute to a progressive decline of the central nervous system in aging and neurodegeneration (Mandemakers et al., 2007). Mitochondrial dysfunction, which can account for all these problems, is commonly involved in neurodegenerative diseases such as Parkinson disease (PD), Alzheimer disease (AD), and Huntington disease (HD) (reviewed in de Moura et al., 2010). However, only little is known about the impact of mitochondria on the pathogenesis of Spinocerebellar Ataxia Type 3 (SCA3). SCA3, also known as Machado-Joseph disease (MJD), is an autosomal dominantly inherited late-onset progressive neurodegenerative disorder which is caused by an expanded CAG repeat and belongs to the group of polyglutamine repeat diseases (reviewed in Riess et al., 2008). The altered protein, ataxin-3, is expressed ubiquitously with strong expression in the central nervous system (Costa et al., 2004). Normally, ataxin-3 localizes in the cytoplasm but in SCA3 patients’ aberrant ataxin-3 also aggregates in the nucleus of a specific subset of neurons. Both, wildtype and polyglutamine-expanded ataxin-3, are also associated with mitochondria (Pozzi et al., 2008; Kristensen et al., 2018). Whether ataxin-3 binds directly to mitochondria or indirectly through interaction partners remains elusive. One possible interaction partner linking ataxin-3 to mitochondria is Parkin, a PD-associated E3 ubiquitin ligase, which is recruited to dysfunctional mitochondria, ubiquitinates outer membrane proteins and targets mitochondria for degradation under stress conditions (Narendra et al., 2008; Durcan et al., 2011). Recent mass spectrometry analyses revealed several new mitochondrial proteins as confirmed or potential interaction partners of wildtype and/ or mutant ataxin-3, including cytochrome C oxidase subunit NDUFA4 (NDUFA4), succinate dehydrogenase (ubiquinone) iron-sulfur subunit (SDHB) and cytochrome C oxidase assembly factor 7 (COA7) (Kristensen et al., 2018). Mitochondrial DNA (mtDNA) deletions were frequently found in SCA3 transgenic mice as well as in most SCA3 patients and were more pronounced in the preclinical stage but not present in healthy individuals or SCA3 mutation carriers (Yu et al., 2009; Kazachkova et al., 2013; Ramos et al., 2015; Raposo et al., 2018). Additionally, the protein mitochondrial genome maintenance exonuclease 1 (MGME1) which is linked to mitochondrial DNA repair was found enriched in ataxin-3 overexpressing HEK293 cells by mass spectrometry analysis. Based on the same methodology, succinate dehydrogenase complex, subunit A (SDHA) and SDHB which are constitute parts of the complex-II of the electron transport chain, were identified as interaction partners of ataxin-3 and therefore, may explain the reduced complex-II activity in SCA3 patient lymphoblast cell lines and cerebellar granule cells from transgenic SCA3 mice (Laço et al., 2012; Kristensen et al., 2018).

It is unclear, if these described alterations are directly linked to full-length wildtype and/ or mutant ataxin-3 or to N- and C-terminal fragments generated by calpain cleavage during the pathogenesis. It was previously shown that the mitochondrial ubiquitin ligase (MITOL) promotes the degradation of N-terminally truncated polyglutamine-expanded ataxin-3 via the ubiquitin-proteasome pathway, attenuating mitochondrial accumulation of the pathogenic ataxin-3 (Sugiura et al., 2011). New insights into the influence of an N-terminally truncated polyglutamine-expanded ataxin-3 fragment to mitochondrial function and health indicate that this truncated form is directly causing increased mitochondrial fission, decreased mitochondrial membrane potential, increased reactive oxygen species and finally increased cell death rates (Hsu et al., 2017). Hsu and coworkers used an artificial N-terminally truncated polyglutamine-expanded ataxin-3 fragment beginning at amino acid position 163 which should represent a truncated fragment derived from cleavage at amino acid position 186 (Haacke et al., 2006). We have published a genetrap mouse model which expresses endogenously a C-terminally truncated mouse ataxin-3 fragment from amino acid 1 to amino acid 259 without harboring the polyglutamine tract (Hübener et al., 2011). Surprisingly, homozygous genetrap mice developed an SCA3 reminiscent neurological phenotype including tremor, weight loss, coordination and balance deficits subsequently leading to premature death which was associated with cytoplasmic aggregate formation, cell death and abnormal response to ER stress (Hübener et al., 2011). Furthermore, our recent study using mass spectrometry demonstrated that ataxin-3 is cleaved by calpains at four positions H187, D208, S256, and G259, and that cleavage at position D208 and S256 demonstrated the most likely pathogenic cleavage fragments in human disease material (Weber et al., 2017). Here, we used mouse embryonic fibroblasts (MEF) generated from the described genetrap mouse model and brain samples from homozygous genetrap mice to analyze the influence of an endogenously expressed N-terminal ataxin-3 fragment (Atx3_1-259_) whose C-terminus is very closely located to an important calpain cleavage site (S256) to study mitochondrial function, biogenesis and mitochondrial mitophagy. The endogenous expression of the truncated N-terminal fragment (Atx3_1-259_) in the murine background resulted in mitochondrial fragmentation and cristae alterations. This was accompanied by a significantly decreased capacity of mitochondrial respiration and decreased mitochondrial membrane potential contributing to an increased susceptibility to apoptosis. Our results indicate that cleavage of full-length ataxin-3 into N- and C-terminal fragments causes important molecular alterations and leads to dysfunctional mitochondria and biogenesis.

## Materials and Methods

### Ethics Statement

This study was carried out in strict accordance with the recommendations presented in the Guide for Care and Use of Laboratory Animals of the University of Tübingen, Germany. The protocols were approved by the Institutional Animal Care and Use Committee (IACUC) of the University of Tübingen, Germany.

### Animal Model

The generation and characterization of the ataxin-3 C-terminally truncated (Atx3_1-259_) mouse model used in this study have been described previously (Hübener et al., 2011, 2012). All animals were housed under standard conditions with a 12 h light-dark cycle, food and water *ad libitum*. For genotyping, primers and conditions were used as described in Hübener et al. (2011). All experiments with mouse brain samples were performed using wildtype, heterozygous and homozygous Atx3_1-259_ mutant mice, experiments with mouse embryonic fibroblasts were only done using wildtype and homozygous Atx3_1-259_ mutant genotypes.

### Cell Culture and Transfection

Mouse embryonic fibroblasts (MEF) with wildtype or Atx3_1-259_ homozygous mutant genotypes were generated from day 12.5 to day 14.5 mouse embryos as previously described (Hübener et al., 2011). Shortly, uterine horns were removed, and each embryo was separated from placenta and surrounding membranes. After removing head and internal organs (used for genotyping), the rest of the embryo was minced into small fragments that were seeded into 25 cm^2^ cell culture dishes. All cells were cultured in Dulbecco’s modified Eagle medium (DMEM; Gibco^®^, Thermo Fisher Scientific) in the presence of 1% penicillin/streptomycin and 10% fetal bovine serum (both Invitrogen) at 37°C and 5% CO_2_. For all analyses only MEF with a passage number lower than 10 were used. The passage number of wildtype and Atx3_1-259_ MEF was identical for all experiments. To analyze endoplasmic reticulum stress, cells were treated with 1 μg/ml tunicamycin (Sigma-Aldrich) for 24 and 48 h.

### Expression Constructs

For transfection of MEF with the pDsRed2-endoplasmatic reticulum vector (Clontech), the pEGFP-LC3 clone (a gift from Tamotsu Yoshimori, Japan), or the pEGFP-N1 ataxin-3 deletion construct (described in Hübener et al., 2011) and pEGFP-N1 ataxin-3 full-length constructs with different polyglutamine length (15Q, 70Q, 147Q), Lipofectamine (Invitrogen) was used as transfection reagent following the manufacturer’s protocol.

### Neuronal Differentiation of Induced Pluripotent Stem Cells

Human fibroblasts from a SCA3 patient (female, 70 CAG repeats in the expanded allele) and a sex-matched health subject were obtained by skin biopsy and reprogrammed to induced pluripotent stem cells (iPSCs) as described in Hayer et al. (2018). Differentiation of iPSCs to iPSC-derived cortical neurons (iCNs) was done as described previously (Shi et al., 2012; Weber et al., 2017).

### Cell-Based Calpain Activation Assay

Activation of endogenous calpains in iCNs was performed as previously described (Weber et al., 2017). Briefly, calpains were activated by incubating cells with 1 μM of the Ca^2+^ ionophore ionomycin (Sigma-Aldrich) and 5 mM CaCl_2_ diluted in Opti MEM^®^ I Reduced Serum Media (Gibco^®^, Thermo Fisher Scientific) for 1 h at 37°C in 5% CO_2_. For negative controls, cells were pre-treated with 10 μM of the calpain inhibitor CI III (carbobenzoxy-valinyl-phenylalaninal) (Merck Millipore) in Opti MEM^®^ I Reduced Serum Media.

### Immunofluorescence Microscopy

Mitochondrial morphology and number of lysosomes were analyzed by live cell microscopy. For this, MEF were cultured in Lab-Tek^®^II chambered coverglasses (Nalge Nunc International). Lysosomes were stained with 50 nM LysoTracker^®^ Red DND-99 and mitochondria with 200 mM MitoTracker^®^ Green FM or MitoTracker^®^ red FM (all Invitrogen) for 15 min at 37°C. Analysis of the number of lysosomes and/ or mitochondrial morphology was performed using an inverted Zeiss Axiovert microscope (Zeiss Plan-Apochromat 63 x/1.4) with an incubation chamber temperature of 37°C. Time lapse pictures were taken with an AxioCamMRm camera (Zeiss) in 5 s intervals for up to 5 min using AxioVision software (Zeiss). The series of pictures was saved uncompressed and analyzed with AxioVision software (Zeiss). Colocalization studies, MEF cells were incubated with 250 nm MitoTracker^®^ red FM for 30 min and afterward fixed with 4% paraformaldehyde for 20 min. After blocking of fixed cells with 10% normal serum, cells were stained with cytochrome C (1:500, Cell Signaling) overnight and the secondary antibody anti-Cy2 (1:300, Jackson ImmunoResearch) for 2 h. DAPI (Invitrogen) was used to stain nuclei. Images were analyzed by Zeiss LSM 510 Confocal Microscope and Zeiss LSM Image Examiner LSM510.

### Quantification of Mitochondrial Morphology

Fluorescence microscopy images were converted to grayscale, inverted to show mitochondria-specific fluorescence as black pixels and the threshold was adjusted to optimally resolve individual mitochondria. Using Image J 1.41o software with a plugin to analyze mitochondrial morphology (Dagda et al., 2009), each single mitochondrion was analyzed for morphological characteristics such as area, perimeter, circularity and major/ minor axis. On the basis of these parameters, the form factor [perimeter^2^/(4π × area)] and the aspect ratio (ratio between the major and minor axes of the ellipse equivalent to the object) were calculated representing mitochondrial elongation and interconnectivity (adapted from Krebiehl et al., 2010).

### Electron Microscopy

Electron microscopy of MEF was performed as previously described (Lundkvist et al., 2004) and followed the description in Hübener et al. (2011).

### Respirometry

Mitochondrial respiration was measured under continuous magnetic stirring either in intact or in homogenized MEF (wildtype and homozygous Atx3_1-259_ cells) with a Clark-type oxygen electrode using a high resolution OROBOROS oxygraph (Haller et al., 1994). For homogenization, MEF were spun down and the pellet was gently homogenized with mild strength by hand for 1 min in cold HBSS buffer (3 × 10^6^ cells/200 μl HBSS consistent of 132 mM NaCl, 5.4 mM KCl, 0.44 mM KH_2_PO_4_, 0.34 mM NaH_2_PO_4_, 0.49 mM MgCl_2_, 0.41 mM MgSO_4_, 10 mM HEPES, 1 mM CaCl_2_, 10 mM pyruvate, pH 7.3). Mitochondrial respiration of intact cells (final concentration 2 × 10^6^ cells/ml) was measured at 37°C in HBSS buffer. Oligomycin (5 μg/ ml), an inhibitor of F_0_F_1_ATPase and FCCP (100 nM), an uncoupler of oxidative phosphorylation were added in the course of the experiment (Gizatullina et al., 2003). Furthermore, mitochondrial respiration in cell homogenates (final concentration 3 × 10^6^ cells/ ml) was measured at 30°C in MMMPK buffer (containing 5 mM MgCl_2_, 120 mM mannitol, 40 mM MOPS, 5 mM KH_2_PO_4_, 60 mM KCl, pH 7.4) by means of multiple substrate inhibitors protocol as described previously (Deschauer et al., 2006) with 10 mM glutamate, 2 mM malate, 1.5 μM rotenone, 10 mM succinate, and 5 μM CAT. Compounds were added with the help of Hamilton syringes through a small hole in the cover of the chamber. The oxygen concentration in the air-saturated medium was determined to be 200 nmol O_2_/ml at 95 kPa barometric pressure. The weight-specific oxygen consumption was calculated from the time derivative of the oxygen concentration (DATGRAPH Analysis software, OROBOROS). Each experiment was repeated eight times independently.

### Mitochondrial Membrane Potential Assessment by Fluorescence-Activated Cell Sorting

Mitochondrial membrane potential (MMP) of MEF generated from homozygous Atx3_1-259_ or wildtype mice was determined by a FACS-based method. For this purpose, 150,000 cells per genotype were seeded as triplicates 48 h prior to performing the FACS measurements. As negative control, cells were treated with 0.5 μM staurosporine for 4 h. Cells were harvested with 2 mM EDTA in phosphate-buffered saline (PBS) and washed twice with PBS. To measure the MMP cells were stained with 200 nM tetramethylrhodamine methyl ester (TMRM, Invitrogen) in PBS for 30 min at 37°C in the dark. After two more washing steps with PBS cells were counted in the fluorescence activated cell sorter (FACS). At least 50,000 cells per sample were analyzed on a CyAnTM ADP apparatus (Beckman Coulter) using the 488 nm argon laser and the PE emission filter (575 nm). Experiments were performed five independent times.

### Quantitative Real-Time PCR

For the detection of differentially regulated genes, RNA was isolated from whole mouse brain using the RNeasy Midi Kit or from MEF using the RNeasy Mini Kit (both Qiagen). RNA quality was validated using an RNA 6000 NanoChip (Agilent Technologies) and complementary DNA synthesis was carried out using the QuantiTect Reverse Transcription Kit (Qiagen). Quantitative real-time PCR was performed with the QuantiTect SYBR green PCR Kit (Qiagen) using the LightCycler^®^ 480 system (Roche Applied Science). Standard curves of each amplified gene were created to calculate PCR efficiency. Hydroxymethylbilane synthase (Hmbs), pyruvate dehydrogenase (lipoamide) beta (Pdhb), succinate dehydrogenase complex, subunit A (Sdha), TATA box binding protein (Tbp), and tyrosine 3-monooxygenase/tryptophan 5-monooxygenase activation protein zeta polypeptide (Ywhaz) were analyzed as reference/ housekeeping genes and were used for normalization. The C_P_-values of the reference and the target genes were obtained with the LightCycler Software 1.5.0 SP3 (version 1.5.0.39). The relative expression levels of all genes were calculated using the mathematical model of Pfaffl (2001). Primer pairs used for polymerase chain reaction amplification are listed in **Supplementary Table S1**.

### Western Blot

Western blot analyses were carried out as previously described (Bichelmeier et al., 2007). Analyses of iCNs from patient-derived iPSCs followed the description in Weber et al. (2017). Antibodies were used at the following dilutions: rabbit anti-cytochrome C (1:1000, #4272), rabbit anti-p62/SQSTM1 (1:500, #5114) (both Cell Signaling), mouse anti-LC3 (0.5 μg/ml, Cat No. 0231, NanoTools), rabbit anti-Dnm1l (1:2000, ab93942), rabbit anti-Fis1 (1:2000, ab96764), rabbit anti-Lamp1 (1:2000, ab24170), rabbit anti-Mfn-1 (1:2000, ab104585), rabbit anti-Mfn-2 (1:2000, ab104632), mouse anti-mitoProfile total OXPHOS (1:2000, ab110413), rabbit anti-Sec61b (1:2500, ab192329), rabbit anti-VDAC (1:2000, ab10527) (all from Abcam), mouse anti-Opa1 (1:1000, Cat No. 612606, BD Biosciences), mouse anti-ataxin-3 (clone 1H9, 1:2000), mouse anti-PGC1α (1:200, ST1202-1) and mouse anti-α-tubulin (1:5000, clone DM1A, CP06) (all Merck Millipore), mouse anti-β-actin (1:5000, A5441, Sigma-Aldrich), goat anti-Ant1/2 (1:1000, sc-9299, Santa Cruz), rabbit anti-ataxin-3 (SA3637, 1:500, Schmidt et al., 1998) as well as peroxidase-conjugated secondary antibodies goat anti-mouse (1:2000, 115-035-003, Jackson Immuno-Research) and donkey anti-rabbit (1:3333, NA934, GE Healthcare Biosciences), or the secondary IRDye antibodies goat anti-mouse 680LT, goat anti-mouse 800CW and goat anti-rabbit 800CW (all 1:10,000, LI-COR Biosciences).

### Detection of Cell Death Rate

Cell death was analyzed in MEF by the Cell Death ELISA (Roche Applied Science) assay, which determines histone-associated DNA fragments, according to manufacturer’s instruction.

### Statistical Analyses

All data were analyzed using Prism 6.0 software, GraphPad. Standard two-way ANOVA (data not matched) to assess the effects of genotype and age and Bonferroni *post hoc* tests were conducted to compare individual genotype effects at individual ages. Data are presented as mean ± SEM. Differences were considered significant if *p* ≤ 0.05.

## Results

### Proteolytic Cleavage of Ataxin-3 in Human Cortical Neurons Leads to Formation of a Specific N-terminal Fragment

**FIGURE 1:**
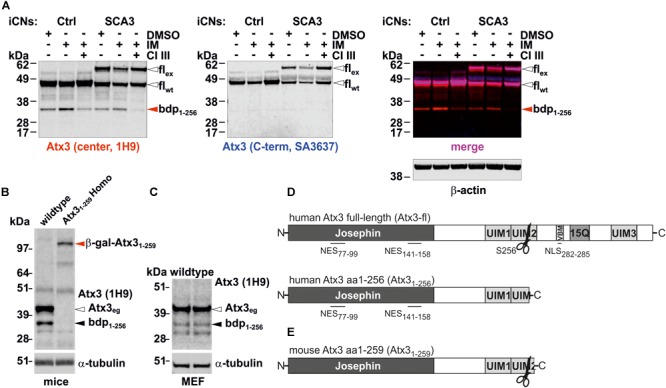
Representation of one of the known calpain cleavage sites at amino acid position S256 in ataxin-3 and the respective breakdown product detected in cortical neurons differentiated from patient and control-derived cell lines as well as in mouse brain and in murine embryonic fibroblasts. **(A)** Western blot analysis of ataxin-3 fragmentation in cortical neurons (iCNs) differentiated from patient-derived iPSCs revealed an N-terminal fragment resulting from calpain cleavage at amino acid S256 (center antibody) after ionomycin (IM) treatment. This effect was abolished by pretreating the cells with calpain inhibitor III (CI III). β-actin is shown as loading control. **(B,C)** Western blot analysis of ataxin-3 fragmentation pattern in wildtype MEF **(C)** and wildtype and Atx3_1-259_ whole brain lysates reveals a breakdown product, derived from cleavage at amino acid position 256 of full-length wildtype ataxin-3 under non-stimulated conditions. In homozygous Atx3_1-259_ whole brain lysates no respective cleavage fragment could be detected, most likely as the fusion protein (β-gal-Atx3_1-259_ is a combination of a cleavage fragment at amino acid position 259 and β-galactosidase) is not further proteolytically processed. As loading control, α-tubulin is shown. **(D)** Schematic illustration of full-length human ataxin-3 (reference isoform, NP_004984.2, Atx3-fl) with a previously identified calpain cleavage site at amino acid position S256, and the localization of the nuclear export (NES) and nuclear import signal (NLS). Cleavage at amino acid S256 lead to an N-terminal fragment (bdp_1-256_), which contains only NES but not the NLS and the polyglutamine tract. **(E)** Schematic illustration of the analyzed murine C-terminally truncated ataxin-3 at amino acid position G259 and the location of the identified calpain cleavage site S256. wt, wildtype; eg, endogenous; ex, expanded; fl, full-length; bdp, breakdown product; β-gal-Atx3_1-259_, fusion protein from β-galactosidase and the Atx3_1-259_ (genetrap approach to generate the mice).

As previously reported, ataxin-3 is cleaved by a group of Ca^2+^-activated endogenous proteases, called calpains. Cleavage at mainly two cleavage sites occasionally leads to the formation of several N-terminal and C-terminal fragments under physiological conditions (Weber et al., 2017). To reinvestigate and confirm this condition, iPSC-derived cortical neurons, generated from an SCA3 patient and a healthy control, were treated with the Ca^2+^- ionophore ionomycin to activate calpains by increasing the intracellular Ca^2+^ levels. Calpain activation by ionomycin led to the reduction of wildtype and polyglutamine-expanded full-length ataxin-3 and the predominant accumulation of an N-terminal, polyglutamine-non-containing fragment (**Figure 1A**), which has recently been identified as an N-terminal breakdown product resulting from cleavage at the amino acid position S256 (Weber et al., 2017). The fragmentation can be also found in whole brain lysates of wildtype mice and wildtype MEF under unstimulated baseline conditions (**Figures 1B,C**). Interestingly, this N-terminal fragment spanning from amino acid 1–256 (Atx3_1-256_) (**Figure 1D**) is only three amino acids shorter at its C-terminal end than the N-terminal fragment (Atx3_1-259_) generated by the genetrap approach in our previously published mouse model (**Figure 1E**). Also of note is, that whole brain lysates of homozygous Atx3_1-259_ mice did not represent the cleavage fragment S256/G259 at around 35 kDa. However, the fusion protein of the respective cleavage fragment and β-galactosidase (generation construct of genetrap mice) can be detected at 110 kDa (**Figure 1B**). Moreover, the N-terminus of ataxin-3 is highly conserved in *Homo sapiens* (reference isoform, NP_004984.2) and *Mus musculus* (isoform 2, NP_083981.2) featuring a completely identical amino acid sequence from amino acid position 1–181, thus including the catalytic Josephin domain as well as the two known nuclear export signals (NES) (compare **Figures 1D,E**). This indicates that the function of the Josephin domain as deubiquitinating enzyme and the calpain cleavage at aa S256 as well as the nuclear export signals (NES) at aa 77–99 and aa 141–158 in *Mus musculus* can be functional active like in humans. Therefore, our mouse model expressing endogenously the murine ataxin-3 fragment Atx3_1-259_ is an excellent model to study the influence of the calpain cleavage at aa S256 and the resulting N-terminal fragment and therefore, the link to SCA3 neuropathology independently from the glutamine stretch *in vivo*.

### Mitochondrial Fragmentation and Cristae Disruption Result in Complex-I and –II Dysfunction

**FIGURE 2:**
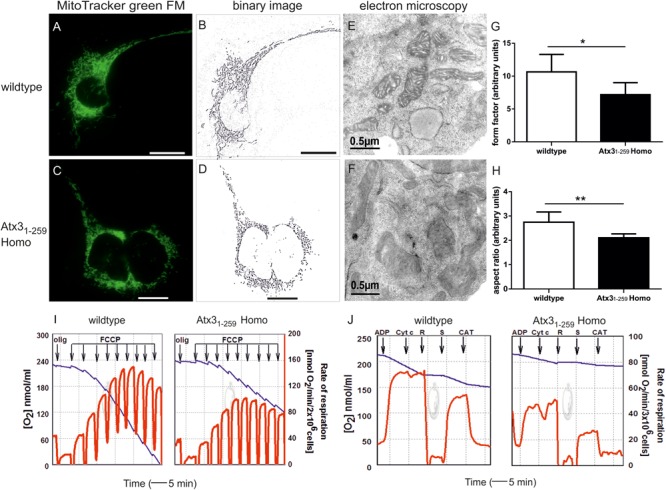
Fragmented mitochondria with disrupted cristae structure and decreased uncoupled respiration in an ataxin-3 mouse model expressing C-terminally truncated ataxin-3 at amino acid position G259 (Atx3_1-259_). **(A–D)** Mitochondrial morphology in living Atx3_1-259_ homozygous and wildtype MEF was analyzed by life cell imaging microscopy (Cell Observer Z1, Zeiss, Germany) at 37°C using ApoTome^®^ optical slices with 0.300–0.350 μm z-stacks. Mitochondria were stained with MitoTracker^®^ green FM and analyzed for area, perimeter, major and minor axis and circularity using Image J 1.41o software. Immunofluorescence analyses revealed a disruption of the tubular and dynamic network of mitochondria in homozygous Atx3_1-259_ MEF **(C,D)** compared to wildtype MEF **(A,B)**. **(E,F)** Electron microscopy showed that increased fragmentation was accompanied by a derangement of mitochondrial cristae structure. **(G,H)** Quantification of mitochondrial morphological changes in homozygous Atx3_1-259_ MEF revealed a significantly reduced degree of mitochondrial branching (form factor, **G**, ^∗^*p* = 0.006) and length (aspect ratio, **H**, ^∗∗^*p* = 0.001) compared to wildtype MEF. Scale bar indicates 20 μm (immunofluorescence) or 0.5 μm (electron microscopy). Representative pictures from 3 independent experiments are shown, in total 45 cells were analyzed. **(I)** Mitochondrial oxygen consumption measured in freshly harvested homozygous Atx3_1-259_ and wildtype MEF in Hank’s medium containing 10 mM pyruvate. Normal endogenous respiration was observed in both wildtype and Atx3_1-259_ MEF without addition of further supplements. Oligomycin-resistant respiration was followed in the presence of 5 μg/ml oligomycin. Stepwise addition of 100 nM FCCP revealed a one third lower activity and less stability against the uncoupler (FCCP) in homozygous Atx3_1-259_ MEF. Starting from the fourth FCCP addition significantly different uncoupled respiration could be observed in Atx3_1-259_ MEF compared to wildtype MEF (^4^*p* = 0.044; ^5^*p* = 0.026, ^6^*p* = 0.024, ^7^*p* = 0.018, ^8^*p* = 0.012, ^9^*p* = 0.011; *n* = 6). **(J)** Complex-I and Complex-II function was analyzed by respiratory measurements in cell homogenates of homozygous Atx3_1-259_ and wildtype MEF using different substrate inhibitors. Measurements were performed for 3 × 10^6^ cells/200 μl in MMMPK buffer with addition of 2 nM ADP, 8 μM cytochrome C (Cyt C), 1.5 μM rotenone (R), 10 mM succinate (S) and 5 μM carboxyatractilate (CAT). Blue line represents oxygen concentration in the oxygraph versus reaction time (left ordinate); red line indicates the first derivative of the oxygen time curve, directly indicating the rate of respiration (right ordinate). This analysis revealed significantly decreased complex-I and complex-II-dependent respiration rates in Atx3_1-259_ homozygous MEF compared to wildtype controls. Typical respirograms from eight independent experiments are shown (Com-I *p* = 0.008; Com-II *p* = 0.033).

Mitochondrial morphological changes were investigated in mouse embryonic fibroblasts (MEF) from wildtype and homozygous Atx3_1-259_ mutant (Atx3_1-259_ Homo) mice by live cell imaging. Significant differences in mitochondrial morphology were found in Atx3_1-259_ homozygous MEF compared to wildtype MEF (**Figures 2A–D**). Mitochondria of Atx3_1-259_ homozygous MEF displayed disintegration of the mitochondrial network and a reduced branching (**Figures 2C,D**) which eventually resulted in disruption of the tubular network compared to wildtype cells (**Figures 2A,B**). Using ImageJ software to quantify mitochondrial branching (form factor, **Figure 2G**, ^∗^*p* = 0.006) and length (aspect ratio, **Figure 2H**, ^∗∗^*p* = 0.001), which both serve as morphological parameters, revealed significantly shorter and less branched mitochondria in Atx3_1-259_ homozygous MEF compared to wildtype MEF. Similar results were obtained by transfecting wildtype MEF with an ataxin-3 specific fragment. The hereby obtained cells remodeled the genetrap mouse-derived Atx3_1-259_ MEF without comprising the C-terminal β-galactosidase component but an EGFP tag. This experiment confirmed that ataxin-3 itself and not the remaining components of the β-galactosidase element influences mitochondrial morphology. Additionally, co-transfection of wildtype MEF with full-length ataxin-3 constructs expressing different polyglutamine lengths (15Q, 70Q, 147Q) revealed fragmented mitochondria in Atx3-fl-70Q and Atx3-fl-147Q compared to wildtype cells expressing a non-expanded ataxin-3 with 15 glutamines (**Supplementary Figure S1**). Electron microscopy showed that the observed increased fragmentation in Atx3_1-259_ homozygous MEF was accompanied by a loss of the double-membrane structure that resulted in a derangement of the inner mitochondrial membrane cristae structure (**Figures 2E,F**).

As disruption of the tubular and dynamic mitochondrial network can result in loss of mitochondrial function, mitochondrial oxygen consumption as measure for mitochondrial respiration was investigated in wildtype and Atx3_1-259_ homozygous MEF. Normal basal respiration was observed in both wildtype and Atx3_1-259_ homozygous cells. Addition of oligomycin, an ATPase-synthase inhibitor, allowed measuring non-phosphorylating respiration (state-4_olig_), which is also similar in both genotypes. Titration with FCCP typically leads to uncoupled mitochondrial respiration followed by respiration inhibition at higher FCCP concentrations. Stepwise addition of FCCP demonstrated significantly decreased FCCP-dependent respiration in intact Atx3_1-259_ homozygous MEF (**Figure 2I**). Statistically, the maximum of uncoupled rates (87.0 ± 23.8 nmol O_2_/min/2 × 10^6^ cells) was already observed at 400 nM FCCP concentration and was about 35% lower in Atx3_1-259_ homozygous MEF compared to wildtype MEF (**Figure 2I** and **Supplementary Table S2**). Hence, also the ratio of maximum uncoupled respiration and state-4_olig_ respiration, a measure for respiratory control (RC), was 25% decreased in Atx3_1-259_ homozygous MEF compared to wildtype MEF (**Supplementary Table S2**). Inhibition of uncoupled respiration was observed in Atx3_1-259_ homozygous MEF at a FCCP concentration of 600 nM compared to 800 nM in wildtype MEF (**Supplementary Table S2**). These results demonstrated that mitochondria of Atx3_1-259_ homozygous MEF have a significantly lower respiratory capacity and are more prone to respiratory uncoupling.

To address whether complex-I (Com-I) or complex-II (Com-II) of the respiratory chain are specifically affected in Atx3_1-259_ homozygous MEF, cell homogenates were investigated by a special substrate inhibitor titration protocol (Deschauer et al., 2006). Hereby, maximum oxidative phosphorylation (state-3_pyr_ respiration) was observed in the presence of complex-I-specific substrates (pyruvate, malate and ADP). Addition of cytochrome C during state-3 respiration subsequently allowed assessing the integrity of the outer mitochondrial membrane. Com-I-dependent state-3 respiration was then inhibited by 1.5 μM rotenone and succinate was added to adjust Com-II-dependent oxidative phosphorylation (state-3_suc_). The rate of state-4_cat_ respiration was measured after inhibiting the ADP/ATP-translocator by carboxyatractyloside. These above described measurements of mitochondrial respiration in cell homogenates represented significantly lower rates of Com-I and Com-II-dependent state-3 respiration in Atx3_1-259_ homozygous MEF compared to wildtype MEF (**Figure 2J**, for Com-I *p* = 0.008; Com-II *p* = 0.033). The ratio of state-3 to state-4 respiration, called mitochondrial respiratory control index (RCI), which indicates the tightness of coupling between respiration and phosphorylation, was significantly lower in Atx3_1-259_ homozygous MEF compared to wildtype controls (wildtype: RCI_I_ = 4.3 and RCI_II_ = 2.8; Atx3_1-259_ Homo: RCI_I_ = 3.6 and RCI_II_ = 2.4). The detected significant reductions of the mass-independent ratios RCI and RC show that decreased capacities of oxidative phosphorylation and, hence, mitochondrial impairment are caused by functional changes and not simply by decreased number of mitochondria.

### Abnormalities in Mitochondrial Morphology and Function Are Not Mainly Caused by Differential Expression of Fission and Fusion Proteins

**FIGURE 3:**
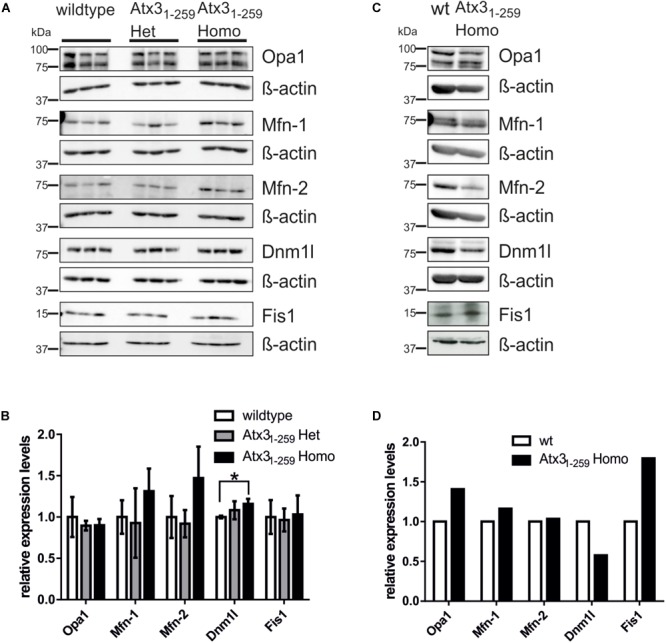
Abnormalities in mitochondrial morphology are not mainly caused by differential expression of fission and fusion proteins. **(A,C)** Western blot analyses in whole brain lysates of 12 months old mice (3 animals per genotype; **A,B**) or MEF lysates (one sample per genotype; **C,D**) represent an equal expression of fission (Dnm1l and Fis1) and fusion proteins (Mfn-1, Mfn-2 and Opa1). β-actin is shown as loading control. **(B,D)** Densitometric quantification revealed significantly increased Dnm1l protein levels in mice homozygous for truncated Atx3_1-259_ compared to wildtype (*p* = 0.028).

To understand the molecular basis of the observed fragmentation, the levels of mitochondrial-shaping proteins were measured. No changes in pro-fusion (Mfn-1, Mfn-2, Opa1) or pro-fission (Fis1, Dnm1l) protein levels were noticed in whole brain lysates of 12 months old phenotypical Atx3_1-259_ homozygous mice (**Figures 3A,B**) or Atx3_1-259_ homozygous MEF (**Figures 3C,D**). However, densitometric quantification revealed significantly increased protein levels of the pro-fission protein Dnm1l in Atx3_1-259_ homozygous mice (*p* = 0.028, **Figure 3B**). Similar results were also found for mRNA levels of the fission and fusion genes in brain lysates of 12 months old phenotypical Atx3_1-259_ homozygous mice compared to wildtype controls (**Supplementary Figure S2A**). Slight mRNA reduction was observed for the fusion proteins Mfn-2 and Opa1 and the fission protein Dnm1l in Atx3_1-259_ homozygous MEF compared to wildtype (**Supplementary Figure S2B**). Moreover, no differences in protein levels of several subunits of the respiratory chain, including complex II-SDHB subunit 30 kDa, complex III subunit Core 2, complex IV subunit I and ATP synthase subunit α (complex V) were found in whole brain lysates of 3 and 12 months old Atx3_1-259_ homozygous mice compared to age-matched wildtype controls (**Supplementary Figures 3A–D**), respectively. This indicates that the above described deficiencies in mitochondrial respiration are not caused by altered expression of respiratory chain proteins.

### Changes in Mitochondrial Morphology and Function Are Linked to Reduced Mitochondrial Membrane Integrity and Higher Rates of Cell Death

**FIGURE 4:**
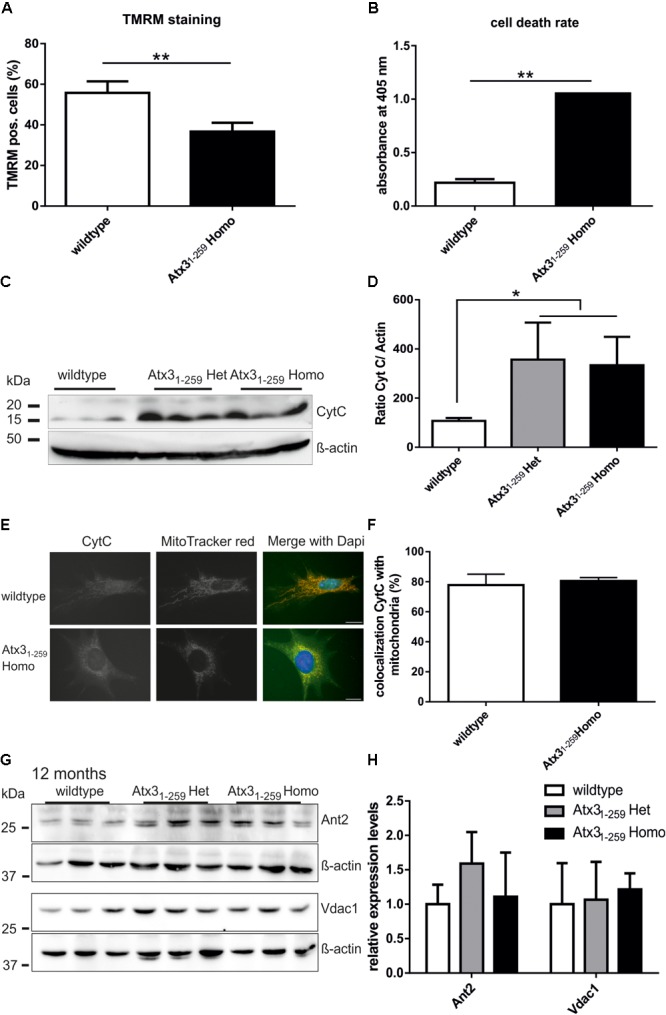
Reduced mitochondrial membrane integrity and higher rate of apoptosis were found in homozygous Atx3_1-259_ MEF. **(A)** Mitochondrial membrane potential (MMP), a marker for mitochondrial membrane integrity, was determined in MEF by TMRM treatment for 30 min at 37°C and subsequent FACS analyses. In five independent experiments a significant reduction of MMP was found in homozygous Atx3_1-259_ MEF compared to wildtype cells (^∗∗^*p* < 0.01). **(B)** Measuring the cell death rate using a cell death-detection kit revealed significantly more apoptosis in homozygous Atx3_1-259_ MEF compared to wildtype MEF (^∗∗^*p* < 0.01, *n* = 3). **(C,D)** As indicator for mitochondrial-associated apoptosis in whole brain lysates of heterozygous and homozygous Atx3_1-259_ mice, the protein level of free cytochrome C was measured in three mice per genotype at the age of 12 months. Densitometric quantification revealed significantly more free cytochrome C in heterozygous and homozygous Atx3_1-259_ mice compared to wildtype controls (^∗^*p* < 0.05). **(E,F)** Immunofluorescence staining of cytochrome C in MEF cells revealed a disrupted mitochondrial network in Atx3_1-259_ homozygous MEF and a good overlap of cytochrome C with mitochondria in both genotypes. **(G,H)** Analyzing protein levels of members of the mitochondrial permeability transition pore (Ant2, Vdac1) in whole brain lysates of 3 mice per genotype also did not identify differences in protein expression at the age of 12 months, which was confirmed by densitometry quantification **(G)**. β-actin is shown as loading control.

Mitochondrial membrane potential (MMP), a measure for healthy and active mitochondria, was investigated by TMRM staining and subsequent fluorescence-activated cell sorting (FACS). The experiment revealed a significant reduction of MMP in Atx3_1-259_ homozygous MEF compared to wildtype MEF (**Figure 4A**, ^∗∗^*p* < 0.01), which is not caused by differential protein expression of members of the mitochondrial permeability transition pore, namely Ant2 and Vdac1 (**Figures 4G,H**). The changes in mitochondrial membrane potential may explain the significantly increased cell death rate observed in Atx3_1-259_ homozygous MEF (**Figure 4B**, ^∗∗^*p* < 0.01). Similar results were found in whole brain lysates of phenotypic Atx3_1-259_ homozygous mice, where the level of free cytochrome C, an indicator for mitochondrial-associated apoptosis, was significantly increased compared to age-matched wildtypes (**Figures 4C,D**, ^∗^*p* < 0.05). Co-immunofluorescence staining of cytochrome C and mitochondria in MEF demonstrated fragmented mitochondria in Atx3_1-259_ homozygous MEF compared to wildtype MEF. Moreover, a clear colocalization of mitochondria and cytochrome C was detected in both genotypes (**Figures 4E,F**).

**FIGURE 5:**
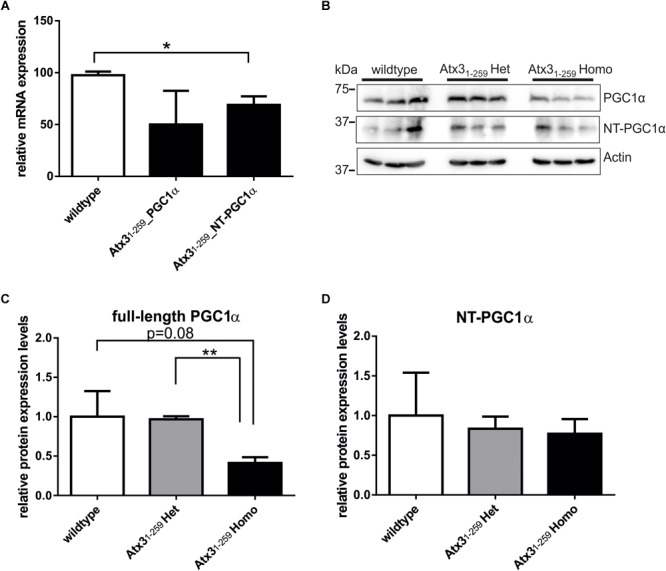
Expression levels of the transcriptional coactivator PGC1α, a regulator of mitochondrial biogenesis, are slightly reduced in homozygous Atx3_1-259_ mice. **(A)** mRNA expression levels of full-length PGC1α and N-terminally truncated PGC1α (NT-PGC1α) are reduced in homozygous Atx3_1-259_ whole brain lysates at the age of 12 months. But statistical analyses revealed that only the differences in mRNA level of N-terminally truncated PGC1α reached significance (*n* = 3; wildtype to Atx3_1-258__PGC1α *p* = 0.16; wildtype to Atx3_1-259__NT-PGC1α ^∗^*p* = 0.032). **(B)** Protein analyses in the same mice (3 per genotype, 12 months of age) demonstrated less full-length PGC1α and similar levels of N-terminally truncated PGC1α in homozygous Atx3_1-259_ mice compared to wildtype mice. **(C,D)** Densitometry quantification revealed no significant differences for the levels of full-length (*p* = 0.081) and N-terminally truncated PGC1α (*p* = 0.932) protein comparing wildtype and homozygous Atx3_1-259_ mice. Significant differences were only found for full-length PGC1α protein levels in homozygous Atx3_1-259_ mice compared to heterozygous Atx3_1-259_ mice (^∗∗^*p* = 0.0025, **C**).

Additionally, analyses of PGC1α, a master regulator of mitochondrial biogenesis, demonstrated slightly decreased mRNA and protein levels in whole brain lysates of Atx3_1-259_ homozygous mice compared to wildtype controls (**Figures 5A–D**). It is controversially discussed in the field whether the full-length or the N-terminally truncated form of PGC1α represents the most promising factor to analyze mitochondrial biogenesis (Cui et al., 2006; Weydt et al., 2006; Johri et al., 2011). In our study, full-length PGC1α showed the strongest downregulation in Atx3_1-259_ homozygous mice compared to wildtype animals. However, densitometric quantification did not reach statistical significance due to the high variability among wildtype animals (**Figures 5A–D**, *p* = 0.081).

### Ataxin-3 Fragmentation Influences Autophagy

As described earlier, mitochondrial dynamics and mitochondrial membrane potential are linked to autophagy and lysosomal impairment. Depolarized mitochondria, which are a substrate for mitophagy, are a consequence of increased mitochondrial fission or decreased mitochondrial fusion. Indeed, mitochondria found in autophagosomes show decreased fusion capability and reduced MMP (Twig and Shirihai, 2011).

**FIGURE 6:**
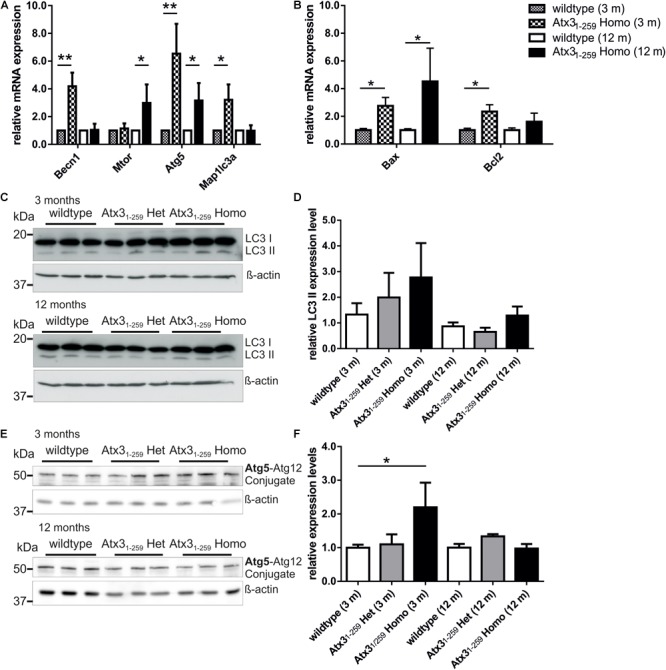
Autophagy is activated in 3 months old but not 12 months old homozygous Atx3_1-259_ mice. **(A,B)** Quantitative real-time PCR revealed differences in expression levels of important autophagic genes and the counter player Bax and Bcl2 in homozygous Atx3_1-259_ whole brain lysates. **(A)** In whole brain lysates of 3 months old homozygous Atx3_1-259_ mice, significantly higher Atg5 and Becn1 mRNA levels (^∗∗^*p* < 0.01) and Map1lc3a mRNA levels (^∗^*p* < 0.05) were found. No changes were found for Mtor mRNA level. Analyzing the same mRNAs at the age of 12 months revealed only a slight upregulation of Atg5 and Mtor (^∗^*p* < 0.05) and no changes in Becn1 and Map1lc3a expression in whole brain lysates of three homozygous Atx3_1-259_ mice compared to wildtype controls. **(B)** Analyzing the mRNA level of pro-apoptotic Bax and anti-apoptotic Bcl2 revealed a upregulation of both in 3 months old homozygous Atx3_1-259_ mice and an overactivation of Bax in 12 months old Atx3_1-259_ homozygous brain lysates (^∗^*p* = 0.047). **(C–F)** Protein expression analyses of Map1lc3a (LC3; **C,D**) and Atg5 **(E,F)** demonstrated a slightly increased Atg5 protein level (^∗^*p* = 0.047) and no differences for the LC3 II protein levels (*p* = 0.149) in 3 months old homozygous Atx3_1-259_ mice. Comparable to mRNA levels, no differences were found for Atg5 and LC3 protein expression at the age of 12 months comparing 3 animals per genotype. β-actin is shown as loading control.

**FIGURE 7:**
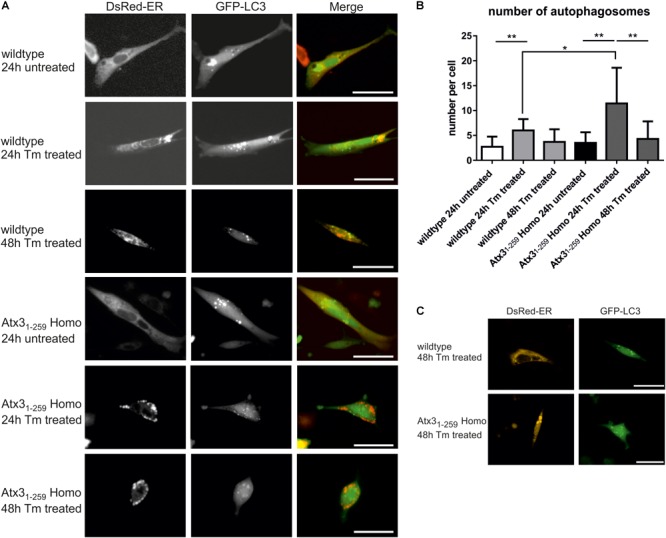
Mild ER stress leads to early autophagy activation but still results in accumulation of unfolded proteins within the ER in homozygous Atx3_1-259_ cells. **(A)** Living wildtype and homozygous Atx3_1-259_ MEF were transfected with pDsRed-ER and pEGFP-LC3 vectors and treated with 1 μg/ml tunicamycin (Tm) for 24 and 48 h. Wildtype cells reacted with producing more autophagosomes after 24 h of treatment and came back to normal steady-state autophagy levels after 48 h of treatment. The ER was not affected. In comparison, homozygous Atx3_1-259_ MEF also started with producing more autophagosomes after 24 h of treatment but at the same time point unfolded proteins started to accumulate within the ER. This accumulation resulted in “bubble-like” DsRed-positive structures found in homozygous Atx3_1-259_ MEF. After 48 h of treatment the accumulation of ER proteins was constantly going on but the number of autophagosomes resembled the number of autophagosomes detected under untreated conditions. Bar indicates 20 μm, represented pictures of three independent experiments is shown. **(B)** Counting the number of autophagosomes in 50 cells from three independent experiments revealed a significantly higher number of autophagosomes in 24 h Tm-treated wildtype and homozygous Atx3_1-259_ cells compared to untreated cells and cells, which were treated for 48 h with tunicamycin. Additionally, the number of autophagosomes was significantly higher in homozygous Atx3_1-259_ MEF compared to wildtype cells after 24 h of treatment (^∗^*p* < 0.05, ^∗∗^*p* < 0.01). **(C)** As a control experiment, cells were transfected with either pDsRed-ER or pEGFP-LC3 and treated with 1 μg/ml tunicamycin for 48 h. Comparable results were seen as demonstrated for double-transfected wildtype and homozygous Atx3_1-259_ cells. Scale bar indicates 20 μm.

To analyze the impact of autophagy on the degradation of fragmented mitochondria found in Atx3_1-259_ homozygous MEF, mRNA and protein expression levels of important autophagic genes/ proteins were measured in MEF and whole brain lysates. mRNA levels of Atg5, Becn1 and LC3 (Map1lc3a) were significantly higher in whole brain lysates of Atx3_1-259_ homozygous mice compared to wildtype controls at the age of 3 months but not at the age of 12 months (**Figure 6A**, ^∗^*p* < 0.05, ^∗∗^*p* < 0.01). On protein level, the amount of LC3 II was slightly but not significantly increased in Atx3_1-259_ homozygous mice at the age of 3 months, and was not affected in 12 months old animals (**Figures 6C,D**, *p* = 0.107). Furthermore, levels of the autophagy receptor protein p62/SQSTM1 in whole brain lysates were not altered between genotypes at 3 and 12 months (**Supplementary Figure S4**). Similarly, Atg5 protein levels (shown as Atg5-Atg12 conjugate) were slightly higher in Atx3_1-259_ homozygous compared to wildtype mice in 3 months old (^∗^*p* = 0.047) but not in 12 months old animals (**Figures 6E,F**). Analyzing the mRNA level of anti-apoptotic Bcl2 and pro-apoptotic Bax revealed an upregulation of both in 3 months old homozygous mice. However, Bax was highly expressed in brain lysates of 12 months old Atx3_1-259_ homozygous mice and, therefore, indicate an activation of apoptotic pathways (**Figure 6B**, ^∗^*p* < 0.05). Combining these data with the increased susceptibility to endoplasmic reticulum stress and ribosomal dissociation described for phenotypic ataxin-3 Atx3_1-259_ homozygous mice in a previous study (Hübener et al., 2011), indicates that autophagy plays a role in the early disease pathogenesis, whereas the chronic stress at late disease stages is triggered by the ER stress response system. To analyze this effect in detail, wildtype and Atx3_1-259_ homozygous MEF were co-transfected with pDsRed-ER and pEGFP-LC3 vectors and mild ER stress was induced by tunicamycin treatment (1 μg/ml) for 24 and 48 h. Both wildtype and Atx3_1-259_ homozygous MEF revealed comparable numbers of autophagosomes under untreated conditions. After 24 h of tunicamycin treatment, both genotypes produced significantly more autophagosomes (^∗∗^*p* < 0.01). Additionally, Atx3_1-259_ homozygous MEF showed ER swelling and “bubble-like” structures as previously observed (Hübener et al., 2011). Treatment for 48 h resulted in autophagosome numbers, which were comparable to numbers observed in untreated cells, in both genotypes. However, ER swelling was constantly visible in Atx3_1-259_ homozygous MEF but was never observed in treated wildtype cells (**Figures 7A,B**). Cells transfected either with pDsRed-ER or pGFP-LC3 and treated with mild ER stress for 48 h demonstrated similar results as shown before by double transfection experiments (**Figure 7C**). ER swelling was also observed in wildtype MEF transfected with polyglutamine-expanded pEGFP-N1 ataxin-3 70Q and 147Q after 48 h of tunicamycin treatment. This was never observed in tunicamycin-treated MEFs transfected with non-expanded ataxin-3 (15Q) or under unstressed conditions (**Supplementary Figure S5**). In conclusion, inducing mild ER stress showed that wildtype cells react to misfolded/ unfolded proteins within the ER by activating the autophagic machinery and thereby maintain normal cell homeostasis. In contrast, overactivation of the autophagy in Atx3_1-259_ homozygous MEF prevented efficient refolding of unfolded/misfolded proteins, which finally resulted in accumulation of unfolded proteins within the ER after 24 h of tunicamycin treatment.

**FIGURE 8:**
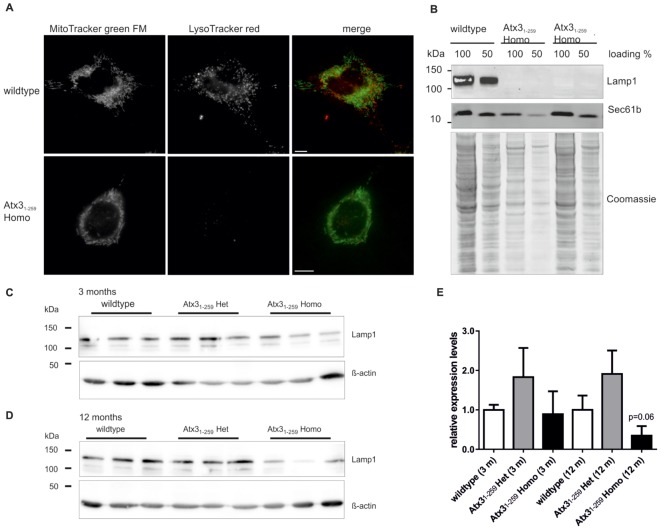
Less lysosomes and abolished expression of lysosomal protein Lamp1 in MEF and slightly reduced expression in whole brain lysates of homozygous Atx3_1-259_ mice. **(A)** Living wildtype and homozygous Atx3_1-259_ MEF were stained with LysoTracker red and MitoTracker green FM and analyzed by life cell microscopy at 37°C using ApoTome^®^ optical slides with 0.300–0.350 z-stacks. Immunofluorescence microscopy demonstrated less lysosomes in homozygous Atx3_1-259_ MEF compared to wildtype controls (15 cells from 3 independent experiments). Bar indicates 10 μm. **(B)** Analyzing the expression level of an important lysosomal protein, namely Lamp1, revealed no Lamp1 expression in homozygous Atx3_1-259_ MEF compared to wildtype controls. Sec61b and coomassie staining are shown as loading control. **(C,D)** Western blot analyses of whole brain lysates of 3 mice per genotype demonstrated no Lamp1 expression differences at the age of 3 months and a reduced Lamp1 level in homozygous Atx3_1-259_ mice compared to wildtype mice at the age of 12 months, without reaching statistical significance in densitometric quantification (**E**; *p* = 0.06).

To further support our data on autophagy, lysosomes were analyzed in Atx3_1-259_ homozygous MEF and Atx3_1-259_ homozygous mice. Counterstaining of MEF with MitoTracker green and LysoTracker red demonstrated significantly fewer lysosomes in Atx3_1-259_ homozygous MEF compared to wildtype MEF (**Figure 8A**) that were also less dynamic (data not shown). Similar results were found by Western blot analysis of the lysosomal marker protein Lamp1, which revealed no Lamp1 protein expression in Atx3_1-259_ homozygous MEF (**Figure 8B**) and less in 12 months old phenotypic Atx3_1-259_ homozygous mice (**Figures 8C–E**) compared to respective wildtype controls.

**FIGURE 9:**
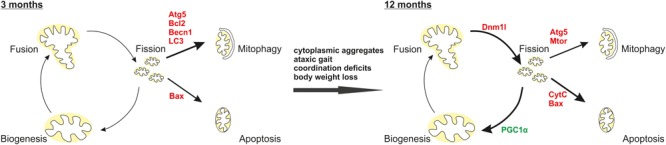
Interplay of mitochondrial homeostasis and mitophagy as well as apoptosis in the pathogenesis of the C-terminally truncated Atx3_1-259_ mouse model. Our results demonstrated that at early disease stages (3 months) the autophagic machinery is activated shown by increased expression of Atg5, Becn1, LC3 and the anti-apoptotic protein Bcl2. After disease onset an impaired mitochondrial homeostasis (increased expression of pro-fission protein Dnm1l, mitochondrial fragmentation, cristae remodeling, reduced mitochondrial membrane integrity) and biogenesis (decreased PGC1α expression) accompanied by an induced apoptosis (increased Bax expression, more cytochrome C release) were found. Red label indicate increased expression, green label demonstrate decreased expression.

In summary, an N-terminal ataxin-3 fragment (Atx3_1-259_) similar to a fragment produced by calpain cleavage (Atx3_1-256_) in human SCA3 patient neurons disrupts mitochondrial function independently from the polyglutamine stretch. Altogether, the above-mentioned results indicate that full-length ataxin-3 has indeed a role in mitochondrial function, biogenesis and homeostasis. Additionally, ataxin-3 fragmentation plays a role in the autophagic lysosomal degradation systems due to misregulation of important autophagic and lysosomal proteins especially at earlier disease stages (**Figure 9**).

## Discussion

Neurons are characterized by particularly high-energy demands to maintain extensive physiological functions. ATP production in neurons mainly relies on functional mitochondria as they cannot switch to glycolysis if oxidative phosphorylation is impaired. Therefore, metabolic defects and loss of body weight, which have been linked to mitochondrial dysfunction, are well described symptoms in neurodegenerative diseases including polyglutamine disorders like HD and SCA3 (reviewed in Weber et al., 2014). Understanding this link between neurodegenerative diseases and mitochondria may pave the way for future therapeutic interventions in neurodegenerative disorders. Both wildtype and polyglutamine-expanded ataxin-3, the disease-causing protein in SCA3, were shown to interact with mitochondria (Pozzi et al., 2008; Kristensen et al., 2018). Up to date, it is unclear, if full-length wildtype and/ or polyglutamine-expanded ataxin-3 or N- and C-terminal fragments generated by proteolytic cleavage events during the pathogenesis are responsible for the observed impaired mitochondrial function and homeostasis. In the last years it was demonstrated that truncated forms and cleavage fragments of ataxin-3 resulted in elevated cytotoxicity and higher aggregation propensity, which in the end lead to neurodegeneration (Ikeda et al., 1996; Haacke et al., 2006; Weber et al., 2017). Last year we identified four calpain cleavage sites in wildtype and polyglutamine-expanded ataxin-3 at amino acid positions H187, D208, S256, and G259 by mass spectroscopy and confirmed the importance of cleavage in position D208 and S256 for human SCA3 pathology (Weber et al., 2017). Hsu et al. (2017) overexpressed an artificial N-terminally truncated wildtype and polyglutamine-expanded ataxin-3 fragment, spanning from amino acid 163 to the proteins C-terminal end, and analyzed its involvement in mitochondrial function. They showed that the ataxin-3 fragment is directly causing increased mitochondrial fission, a decreased mitochondrial membrane potential, increased reactive oxygen species and finally increased cell death rates (Hsu et al., 2017). We generated and characterized an ataxin-3 mutant mouse model using the genetrap approach. This mouse model endogenously expresses C-terminally truncated murine ataxin-3, which ends at the recently identified calpain cleavage site G259 and, moreover, resembles a prominent cleavage fragment of human SCA3 pathology resulting from calpain cleavage at amino acid position S256 (Hübener et al., 2011; Weber et al., 2017). Although the endogenous murine ataxin-3 misses the polyglutamine repeat, mice expressing the truncated protein develop neurological symptoms with gait ataxia, coordination and balance problems, tremor, weight loss, and premature death. Regarding molecular neuropathology, the model is characterized by neuronal cytoplasmic inclusion bodies, neuronal cell death and increased susceptibility to endoplasmic reticulum stress including ribosomal dislocation (Hübener et al., 2011). To study mitochondrial (dys)function and autophagic impairment, brain lysates and MEFs obtained from wildtype and homozygous Atx3_1-259_ mutant mice were analyzed. However, there is knowledge that fibroblast cultures are not able to mimic the brain region-specific patterns of morphological alterations in MJD. Due to their nature, these primary cells are unable to form neuronal structures as synapses and as synaptic mitochondria are known to behave differently from non-synaptic mitochondria, respective effects cannot be investigated in this model (Lai et al., 1977; Brown et al., 2006). Nevertheless, MEF isolated from animal models of neurodegenerative disorders are often used as *ex vivo* model to study the influence of the mutation without the need of an artificial overexpression. Additionally and despite their limitations, fibroblast cultured from human disease patients and the respective animal models are often used along with biomaterials isolated from patients/ animal models as these cells are easy to generate and to handle (Auburger et al., 2012; Lei, 2013).

Additional to the fact that *in vivo* expression of the N-terminal ataxin-3 cleavage fragment lacking the polyglutamine stretch led to a phenotype reminiscent of SCA3 pathology, we found in the present study comparable mitochondrial deficits as earlier shown for N-terminally truncated ataxin-3 fragments with expanded polyglutamine tract (Hsu et al., 2017). Both studies demonstrated fragmented mitochondria, changes in fission and fusion, decreased mitochondrial membrane potential and higher cell death rate. Additionally, we found an impaired mitophagy with an activation of autophagy only in early pathogenesis combined with a reduced lysosomal activity. Changes in mitochondrial morphology are already linked to pathogenesis of other polyglutamine diseases, especially shown for HD. Here, HeLa cells overexpressing polyglutamine-expanded huntingtin as well as lymphoblast’s of HD patients revealed fragmented mitochondria and disrupted cristae structure (Costa et al., 2010). Costa and colleagues demonstrated that mitochondrial fragmentation is associated with increased activity of the pro-fission protein Dnm1l, which is regulated by the calcium-dependent phosphatase calcineurin (Costa et al., 2010). Additionally, a direct interaction of mutant huntingtin and Dnm1l was shown, which influences the GTPase activity of Dnm1l (Song et al., 2011; Shirendeb et al., 2012). Similar to these results, we found fragmented mitochondria and a disrupted cristae structure in our C-terminally truncated Atx3_1-259_ homozygous mice. Additionally, the pro-fission protein Dnm1l was significantly increased at the protein level in Atx_1-259_ homozygous mice, whereas the other mitochondria shaping proteins were not differentially regulated. So far, our study showed that ataxin-3 cleavage fragments influence mitochondrial morphology and function independent of its polyglutamine tract.

In order to learn whether ataxin-3 fragmentation also influences mitochondrial biogenesis, the expression of PGC1α, peroxisome proliferator-activated receptor-γ coactivator 1α, a master regulator of energy homeostasis and mitochondrial biogenesis, was investigated. Differential expression of PGC1α is known to influence mitochondrial dysfunction in HD, but previous studies also linked regulation of PGC1α activity to general aging and other neurodegenerative diseases including AD and PD (reviewed in Austin and St-Pierre, 2012). As ataxin-3 can regulate nuclear gene expression (Evert et al., 2006), mRNA as well as protein levels of full-length and N-terminally truncated PGC1α were analyzed in this study. Whether full-length or N-terminally truncated PGC1α affects mitochondrial biogenesis is still under discussion (Cui et al., 2006; Weydt et al., 2006; Johri et al., 2011). We found significantly reduced mRNA levels of N-terminally truncated PGC1α in Atx3_1-259_ homozygous mice, whereas changes in the level of full-length PGC1α did not reach statistical significance due to the high variability within one genotype. This variability may be explained by different metabolic situations, which have been shown to fine-tune PGC1α expression (Cantó and Auwerx, 2009). At protein level, no expression differences were found for N-terminally truncated PGC1α, whereas the full-length PGC1α is clearly reduced in homozygous Atx3_1-259_ C-terminally truncated mice. In addition, Atx3_1-259_ homozygous mice demonstrated a drastic reduction of body weight (up to 20–30 g of total weight) and reduced amounts of dopamine in the striatum at 12 months of age, the beginning of their neurological phenotype (Hübener et al., 2011, 2012). Reduction of dopamine content, which resulted from selective loss of dopaminergic neurons and increased dopamine levels, has also been reported for HD models steadily overexpressing PGC1α (Ciron et al., 2012).

While the exact correlation between disruption of the mitochondrial network and mitochondrial function and biogenesis is still a matter of debate in the case of HD (reviewed in Costa and Scorrano, 2012), complex-II impairment emerges as key event of mitochondrial dysfunction in SCA3 and has been observed in different SCA3 cell and animal models as well as in peripheral cells of SCA3 patients (Laço et al., 2012). Additionally, mass spectroscopy analyses of polyglutamine-expanded ataxin-3 identified SDHA and SDHB, which are constitute part of the complex-II, as potential interactors (Kristensen et al., 2018). Concordantly, the fragmented mitochondrial network observed in the SCA3 model analyzed in the present study came along with reduced complex-I and complex-II activities and reduced uncoupled respiration upon FCCP treatment. These respiratory deficiencies finally resulted in impaired mitochondrial membrane integrity and an increased rate of mitochondrial-associated apoptosis. Cytochrome C release from the mitochondrial intermembrane space into the cytosol, which is a key event in triggering intrinsic apoptosis, depends on two changes of mitochondrial morphology influenced by Dnm1l, i.e., mitochondrial fragmentation and cristae remodeling (Frank et al., 2001; Scorrano et al., 2002). Indeed, we detected increased rates of cytochrome C release in brain lysates of our Atx3_1-259_ homozygous mice and reduced mitochondrial membrane integrity. Moreover, a higher susceptibility to apoptosis was confirmed in homozygous Atx3_1-259_ MEFs. Earlier studies on neuronal SK-N-SH cells expressing polyglutamine-expanded ataxin-3 demonstrated increased cytochrome C levels under basal conditions in combination with a significantly decreased expression of the anti-apoptotic protein Bcl2 (Tsai et al., 2004). Bcl2 is localized in the outer membrane of mitochondria, where it plays an important role in promoting cellular survival and inhibiting the actions of pro-apoptotic proteins like Bax. The mRNA levels of Bcl2 in our C-terminally truncated Atx3_1-259_ homozygous mice revealed an increased expression of Bcl2 at early disease stages, but no differences at 12 months where we found increased cytochrome C levels as described by Tsai et al. (2004). Additionally, the mRNA levels of the pro-apoptotic protein Bax were increased in Atx3_1-259_ homozygous mice at the age of 3 and 12 months comparable to a study from Chou et al. (2011). Chou and coworkers demonstrated that polyglutamine-expanded ataxin-3 led to upregulation of the pro-apoptotic proteins Bax and PUMA and caused apoptotic death via enhancing the phosphorylation and transcriptional activity of p53 (Chou et al., 2006, 2011). Recently, evaluation of the activation of the intrinsic apoptotic pathway by determining the Bcl2/Bax ratio demonstrated lower values in SCA3 mutation carriers as well as in pre-ataxic subjects compared to controls. In combination with the also analyzed accumulation of mitochondrial mtDNA deletions, it was hypothesized that apoptosis is initiated during the pre-ataxic stage, which might lead to an upregulation of cell death in subsequent disease stages (Raposo et al., 2018).

Mitochondrial deficiencies, as observed in the herein analyzed Atx3_1-259_ C-terminally truncated mice, can lead to intrinsic apoptosis as described above but may also account for increased mitophagy, a process which selectively degrades mitochondria by autophagy (Twig and Shirihai, 2011). Induction of mitophagy is tightly linked to mitochondrial quality control. Depending on the dimension of mitochondrial damage, fragmented mitochondria either fuse back to a functional network or undergo mitophagy. Dysregulation of autophagy has been demonstrated in SCA3 patients’ post-mortem brain samples. In the cerebellum and oculomotor nucleus of SCA3 patients, autophagic proteins such as autophagy-related gene (Atg) protein ATG-7, -12, -16L2 and microtubule-associated proteins 1A/1B light chain 3A and B (MAP1LC3A/B) were significantly increased (Sittler et al., 2018). In the present study, investigation of Map1lc3a and the Atg12-Atg5 conjugate, two factors essential for autophagosome formation, revealed an increased autophagy at early stages of the disease but not at stages when first neurological symptoms appear in the Atx3_1-259_ homozygous mice. Inducing mild ER stress in MEF by tunicamycin treatment resulted in an increased activation of autophagy in Atx3_1-259_ MEFs after short treatment periods and in increased ER stress response upon continued treatment. This crosstalk between autophagy and ER stress was already described for neurodegenerative diseases like HD (Vidal and Hetz, 2012; Vidal et al., 2012). Upon autophagosome maturation, autophagosomes fuse with lysosomes containing different enzymes, which are required for degradation of the autophagosomal content. Hence, normal lysosomal function and organization are essential for autophagy and lysosomal disturbances have been associated with neurodegeneration (reviewed in Appelqvist et al., 2013). In 2016 a direct link between mitochondrial dysfunction and disruption of structure and function of lysosomes was demonstrated in cells and brains (Demers-Lamarche et al., 2016). Mitochondrial dysfunction is characterized by mitochondrial fragmentation, decreased mitochondrial ATP and increased ROS production, which all can potentially influence lysosomal structure and activity. Demers-Lamarche and colleagues demonstrated that lysosomal impairment is mainly caused by increased ROS production and linked to large Lamp1-positive vacuoles (Demers-Lamarche et al., 2016). The present study also detected lysosomal disturbances in form of a decreased number of lysosomes and impaired lysosomal dynamics in Atx3_1-259_ MEF. Lamp1, a marker for late-endosomes, lysosomes and autolysosomes, is known to be involved in lysosome biogenesis and autophagy (Eskelinen, 2006), and differential expression as well as colocalization with polyglutamine-containing aggregates in HD were already reported (King et al., 2008; Zheng et al., 2010). In our study, the reduction in the number of lysosomes was accompanied by a reduced expression of Lamp1 in brain samples of phenotypic Atx3_1-259_ homozygous mice.

Summing up, this study shows that an N-terminal S256 ataxin-3 cleavage fragment impairs mitochondrial morphology, function and homeostasis as well as disturbs proper clearance of impaired mitochondria via mitophagy. Interestingly, together with Hsu et al. (2017) we could demonstrate that both N- and C-terminal fragments of ataxin-3 generated by calpain cleavage may lead to a mitochondrial phenotype independent of the expression of the polyglutamine tract. Therefore, further research on the influence of cleavage-derived fragments in different areas as well as *in vitro* and *in vivo* models that are relevant for SCA3 pathogenesis is demanded to elucidate whether the function of full-length ataxin-3 is directly compromised by the pathologically elongated polyglutamine stretch or by consequently occurring ataxin-3 fragments.

## Author Contributions

TH, CP, JW, CF, SD, JM handled animal studies, molecular analyses, and analyzed the data. FG performed the mitochondria respiration studies. GK and RK helped with FACS analyses and live cell microscopy. HW performed the electron microscopy. SNH, SH, and LS generated and characterized iPSCs and iCNs from SCA3 patients and controls. OR and JH designed the experiments and oversaw the progression of the study. TH, CP, JW, and JH drafted the paper. All the authors read and approved the final manuscript.

## Conflict of Interest Statement

The authors declare that the research was conducted in the absence of any commercial or financial relationships that could be construed as a potential conflict of interest.
