# Supplementary material for: Mitochondrial Morphology, Function and Homeostasis Are Impaired by Expression of an N-terminal Calpain Cleavage Fragment of Ataxin-3
Source: Front Mol Neurosci. 2018 Oct 10;11:368. doi: 10.3389/fnmol.2018.00368 (PMC6192284; doi:10.3389/fnmol.2018.00368)
Supplement: Supplementary file 1 [file Data_Sheet_1.PDF]

Supplement material

Supplementary Table S1: Primer sequences used for quantitative real-time PCR analyses

	**forward (5´- 3´)**	**reverse (5´- 3´)**
Atg5	aagtctgtccttccgcagtc	tgaagaaagttatctgggtagctca
Bax	gtgagcggctgcttgtct	ccatcttcttccagatggtga
Bcl2	ctacgagtgggatgctggag	aggggcctgagaggagac
Becn1	atgcaggtgagcttcgtgt	gcctgggctgtggtaagtaa
Dnm1l	gttgcccgtgacaaatgaa	atcagcaaagtcggggtgt
Fis1	atatgcctggtgcctggtt	catagtcccgctgttcctct
Hmbs	cccatgtgccttcagtcc	ccctcatctttgagccgttt
Map1lc3a (LC3)	catgagcgagttggtcaaga	accatgctgtgctggttg
Mfn-1	ccgatggagataaagcctacc	caagagggcacattttgctt
Mfn-2	tccctctcaagcactttgt	ccagttctgtgttcctgtgg
Mtor	tggacacaaacaaggaagacc	cagagtccacttttcacagca
Opa1	ttcgggaacatttaacacca	cctttcctttgggttctgtc
Pdhb	gtagaggacacgggcaagat	tgaaaacgcctcttcagca
PGC1α	gatggcacgcagccctat	ctcgacacggagagttaaaggaa
NT- PGC1α	tgccattgttaagaccga	ggtcactggaagatatgg
Sdha	gcagcacagggaggtatca	ctcaaccacagaggcagga
Tbp	tctattttggaagagcaacaaagac	gaggctgctgcagttgcta
Ywhaz	gaggaaaccccgtgtctg	ccttctgcaccagctcattt

Supplementary Table S2: Mitochondrial properties in wildtype and homozygous $Atx3_{1\text{-}259}$ MEF in the presence of oligomycin and FCCP. Respiratory control (RC) demonstrate the ratio of maximum uncoupled respiration and state-4_{olig}. Summary of eight independent experiments with $2x10^6$ cells used in each experiment.

	Basal Respiration	State-4_{olig}	FCCP 100 nM	FCCP 200 nM	FCCP 400 nM	FCCP 600 nM	FCCP 800 nM
wildtype	42.0±10.6	14.5±2.5	50.5±7.0	82.6±13.1	125.6±26.3	132.8±31.8	116.5±27.7
RC_{WT}						9.16	
$Atx3_{1\text{-}259}$ Homo	35.0±18.5	12.7±2.6	39.5±11.4	62.8±16.5	87.0±23.8	83.2±21.9	71.5±18.5
$RC_{Atx31\text{-}259\ Homo}$					6.85		

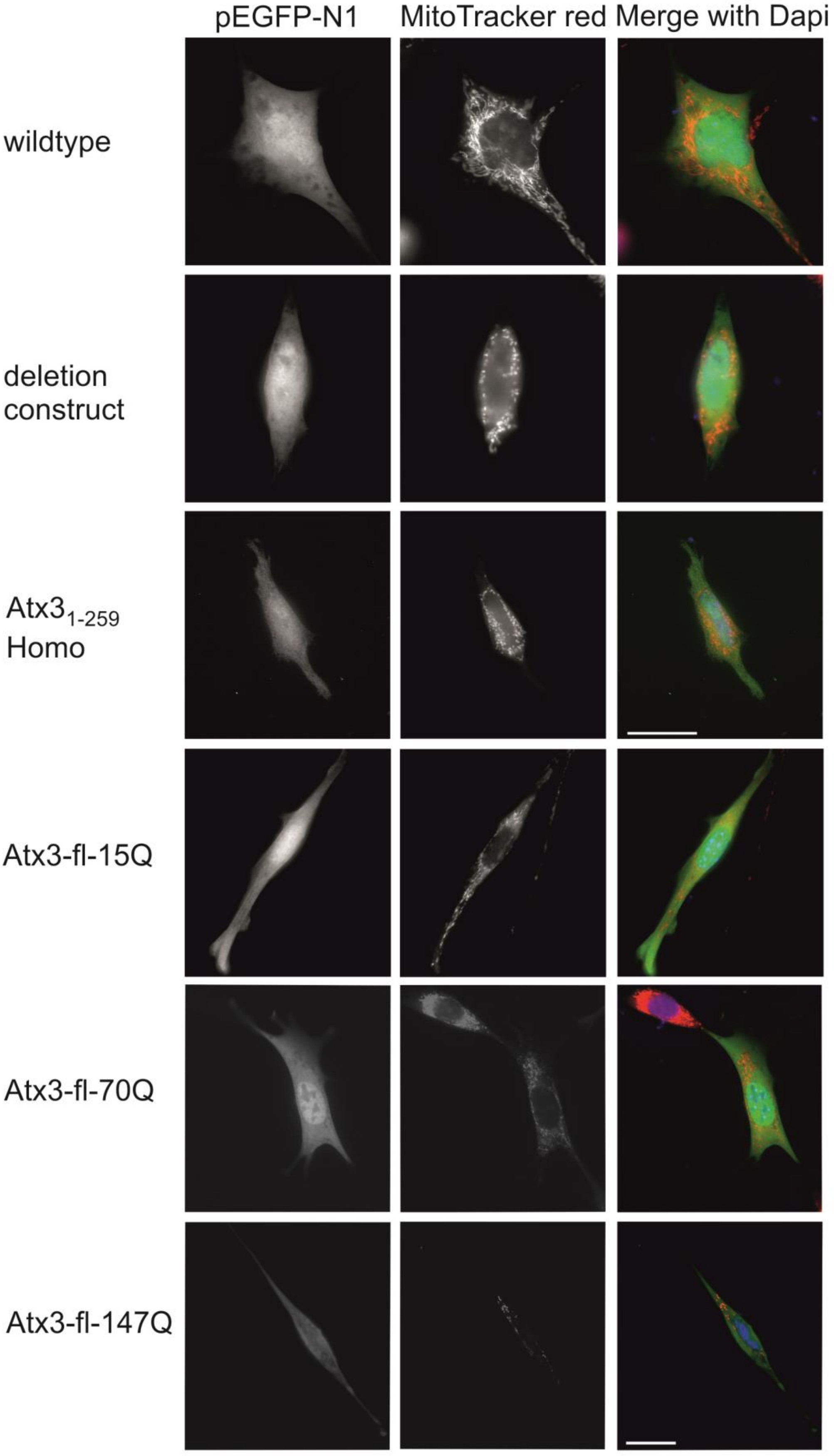

Supplementary Figure S1: Overexpression of C-terminally truncated ataxin-3 ($Atx3_{1-259}$) in wildtype MEF demonstrated fragmented mitochondria similar as seen in $Atx3_{1-259}$ Homo MEF. The exact copy of the Ataxin-3 genetrap mouse model, but which lacks the PT1βgeo

vector including lacZ and neomycin-resistance cassette used to generate the mouse model (Hübener et al., 2011), called deletion construct was cloned into a pEGFP-N1 vector and transfected to wildtype MEF to confirm that the C-terminally truncated ataxin-3 itself lead to mitochondria abnormalities. As control experiment, wildtype MEF and $Atx3_{1\text{-}259}$ homozygous MEF were co-transfected with an empty pEGFP-N1 vector. Additionally, wildtype MEF were co-transfected with pEGFP-N1 full-length (fl) ataxin-3 with 15, 70 and 147 glutamines. Immunofluorescence analyses using MitoTracker® red FM revealed fragmented mitochondria in MEF transfected with the deletion construct ($Atx3_{1\text{-}259}$) and pEGFP Atx3-fl-70Q and pEGFP Atx3-fl-147Q similar to the fragmented mitochondria seen in $Atx3_{1\text{-}259}$ homozygous MEF. Wildtype MEF co-transfected with empty pEGFP or pEGFP Atx3-fl-15Q demonstrated a normal mitochondrial network. Scale bar indicates 20 μm.

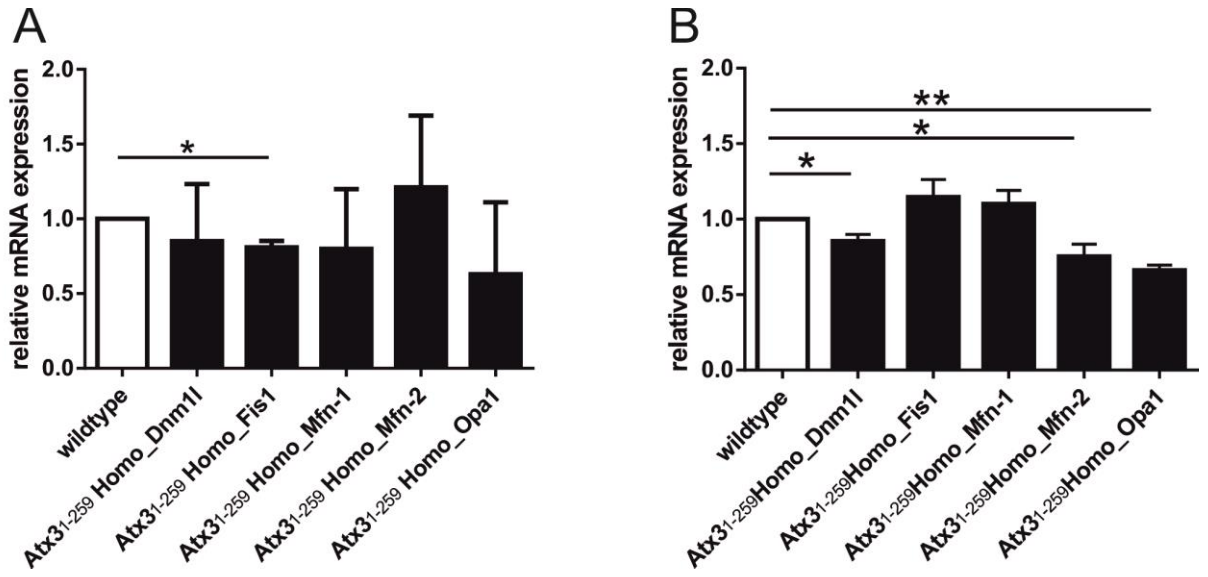

Supplementary Figure S2: mRNA studies of fission and fusion genes revealed significantly reduced Fis1 expression in $Atx3_{1-259}$ homozygous mouse brain and significantly lower Mfn-2, Opa1 and Dnm1l mRNA expression in $Atx3_{1-259}$ homozygous MEF

(A) Quantitative real-time PCR of different fission and fusion genes in whole brain lysates of 12 months old $Atx3_{1-259}$ homozygous mice compared to wildtype controls revealed no expression differences except of Fis1 in which a slightly decreased mRNA level was detected (* $p = 0.03$). **(B)** qRT-PCR in MEF isolated from $Atx3_{1-259}$ homozygous mice compared to wildtype controls revealed a significant lower mRNA expression of the pro-fusion proteins Mfn-2 and Opa1 and the pro-fission protein Dnm1l. No significant differences were found for Fis1 and Mfn-1 (* $p<0.05$, ** $p<0.01$). n = 3

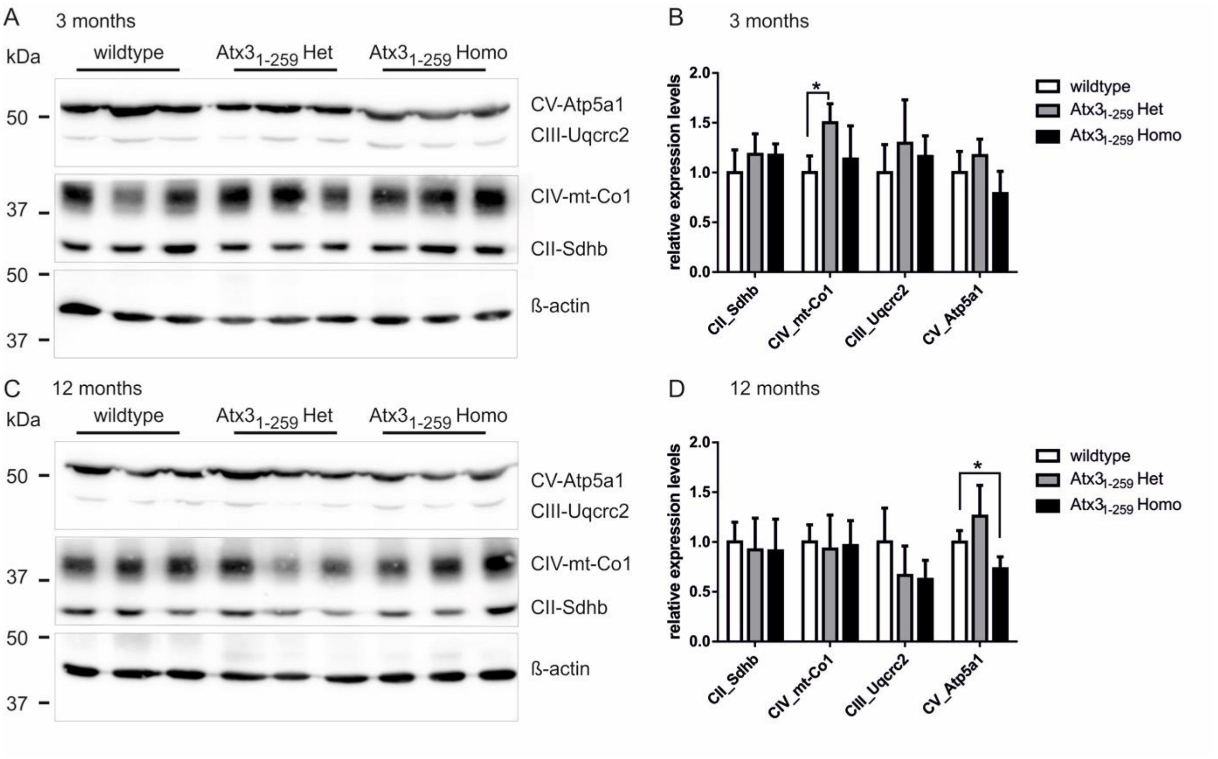

Supplementary Figure S3: Only slight changes in protein levels of respiratory chain proteins.

(A-D) Western blot analyses of selected respiratory chain proteins at the age of 3 **(A,B)** and 12 months **(C,D).** Quantification demonstrated significant difference between wildtype and homozygous $Atx3_{1\text{-}259}$ mice for complex-V (Atp5a1) at the age of 12 months (**D**, * p = 0.048) and between wildtype and heterozygous $Atx3_{1\text{-}259}$ ($Atx3_{1\text{-}259}$ Het) mice for complex-IV (mt-Co1) at the age of 3 months (**B**, * p = 0.026). Shown and quantified are whole brain lysates from 3 mice per genotype and age, β-actin is shown as loading control.

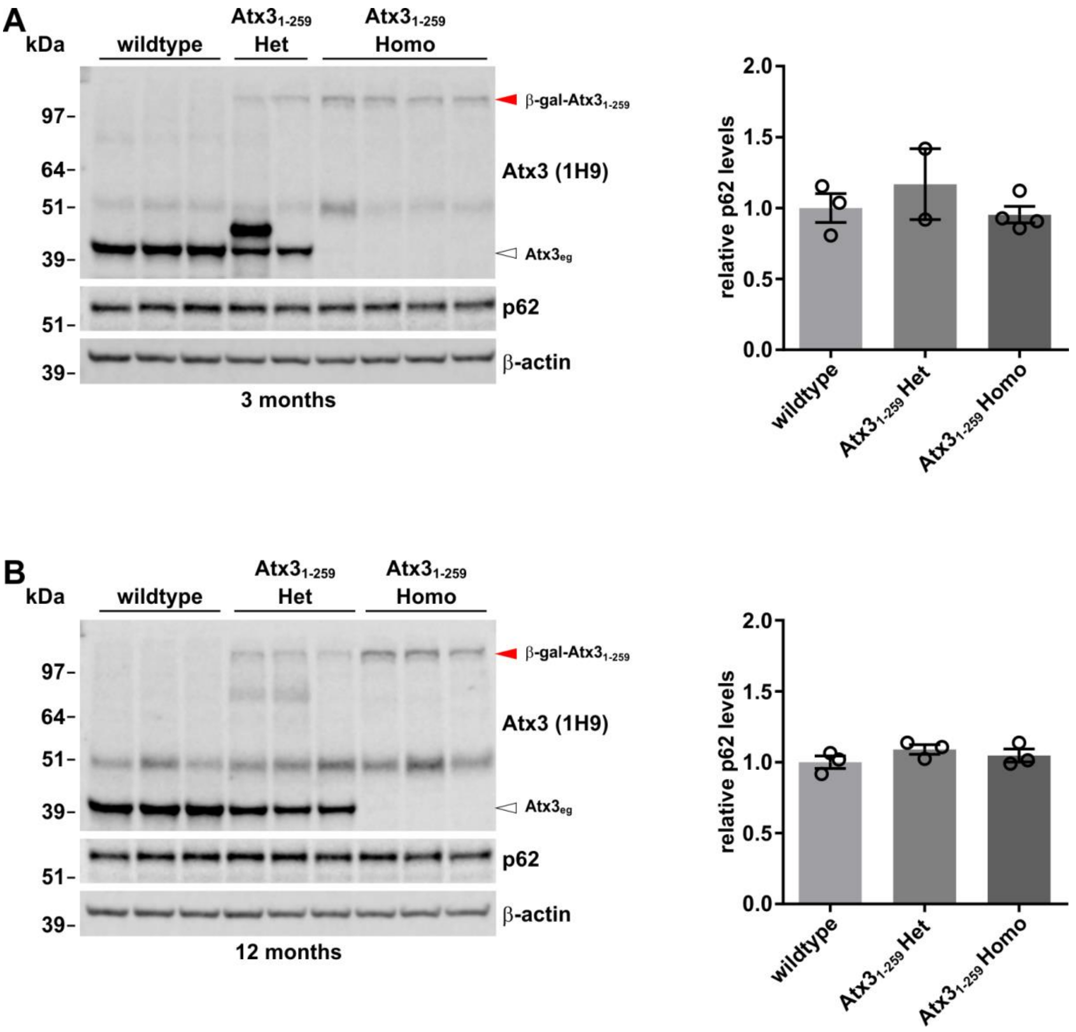

Supplementary Figure S4: No changes in protein level of the autophagy cargo protein p62/SQSTM1.

(A-D) Western blot analyses of p62 at the age of 3 **(A)** and 12 months **(B).** Quantification demonstrated no expression difference between wildtype and homozygous $Atx3_{1\text{-}259}$ mice. Immunoblotting with ataxin-3 confirmed the genotype of analyzed mice. Shown and quantified are whole brain lysates from 3 mice per genotype (at 3 month 2 heterozygous and 4homozygous mice) and age, β-actin is shown as loading control.

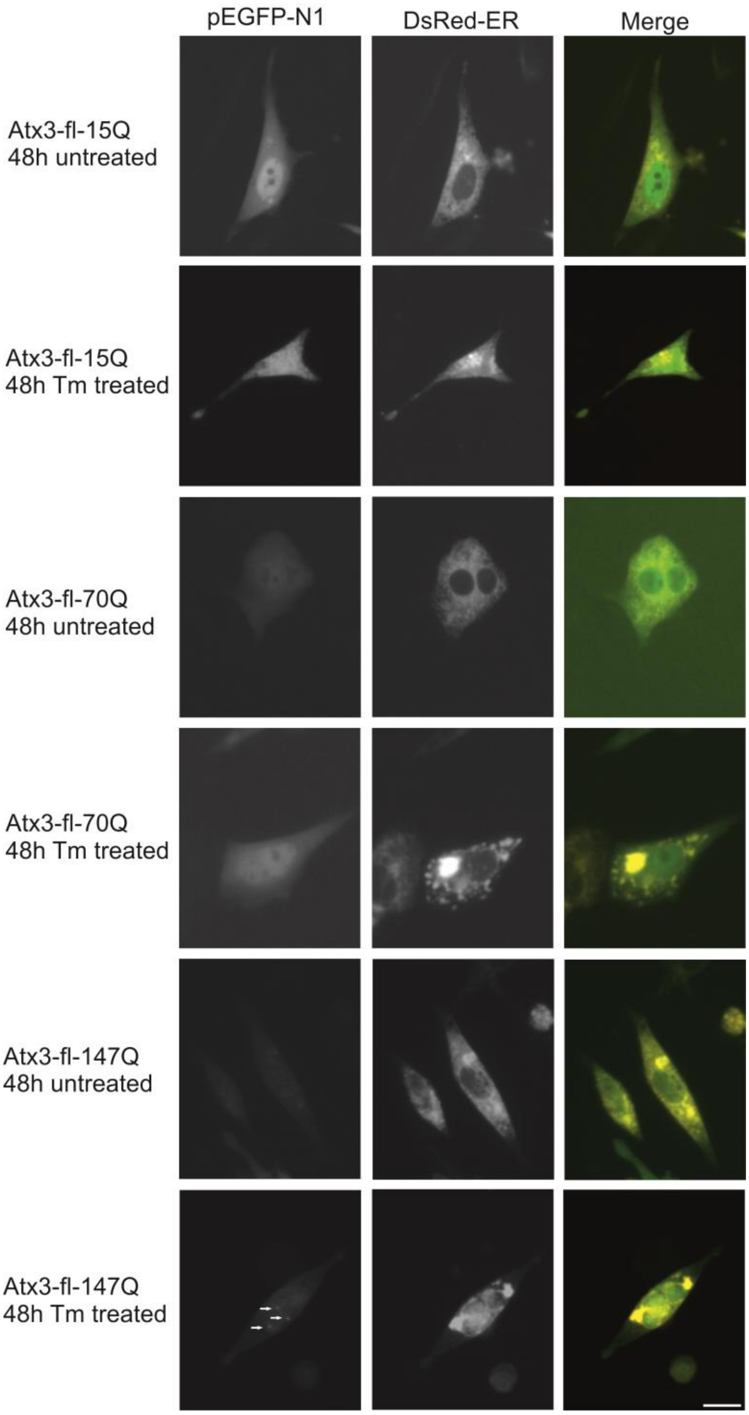

Supplementary Figure S5: Mild ER stress leads to accumulation of unfolded proteins within the ER in polyglutamine expanded ataxin-3 with 70 and 147 glutamines

Living wildtype MEF were transfected with pEGFP-N1 ataxin-3 constructs with different polyglutamine length (15Q, 70Q, 147Q) and treated with 1 µg/ml tunicamycin (Tm) for 48 hours. Wildtype MEF transfected with expanded polyglutamine tract (70Q and 147Q) demonstrated a “bubble-like” DsRed-positive structure after 48 hours of Tm treatment, which was never found in treated wildtype MEF transfected with non-expanded ataxin-3 (15Q). In untreated conditions a normal ER structure were found in all wildtypes MEF transfected with the different polyglutamine length (15, 70 and 147Q). Additionally, in Tm treated MEF with 147glutamines GFP-positive accumulations which seems to be first aggregates were detected

in around 15% of all analyzed cells (indicated by arrow). Scale bar represents 20µm, represented pictures of three independent experiments is shown.
